# Communication Preferences of School-Age Children with Cochlear Implants in Multilingual Educational Settings: Implications for Inclusive Education and Public Health

**DOI:** 10.3390/ijerph22111699

**Published:** 2025-11-11

**Authors:** Muhammed Ayas, Marwa Madi

**Affiliations:** 1 College of Health Sciences, University of Sharjah, Sharjah P.O. Box 27272, United Arab Emirates; 2 Sharjah City for Humanitarian Services, Sharjah P.O. Box 5796, United Arab Emirates

**Keywords:** cochlear implants, bimodal communication, multilingualism, adolescents, inclusive education, public health

## Abstract

**Background:** School-age children with cochlear implants (CIs) navigate academic and social settings by adopting varied communication strategies. Understanding these preferences and their determinants is essential for inclusive education and equitable public health. Evidence from multilingual contexts remains limited. **Objective:** The aim of this study was to investigate the communication preferences among school-age children with CI and the influence of social adaptability, home language, and CI duration. **Methods:** A cross-sectional study was conducted with 32 CI user school-age children (mean age = 13.4 years) at Sharjah City for Humanitarian Services. A structured questionnaire assessed communication mode, adaptability, comfort, and effectiveness. Quantitative associations were tested with Chi-square or Fisher’s exact tests; Pearson’s correlation examined links with CI duration. Qualitative responses were thematically analysed. **Results:** Most school-age children with CI preferred bimodal communication (spoken and sign). The primary spoken language used in the household was associated with communication preferences across settings (*p* ≤ 0.031). Gender differences appeared in family communication (*p* = 0.036). Longer CI duration correlated with greater spoken-language comfort (r = 0.32; *p* = 0.038). Self-reported adaptability was high but not significantly associated with preferences. **Conclusions:** School-age children with CI in multilingual environments predominantly adopt bimodal communication, shaped by sociocultural and linguistic contexts. Recognising bimodal use as normative supports bilingual education, family-centred care and public health strategies promoting equity and participation.

## 1. Introduction

Effective communication is essential for academic achievement, psychosocial development, and social participation in children with hearing loss. For school-age children with cochlear implants (CI), this process is particularly complex as they must navigate diverse communicative environments and adjust between spoken and signed modalities depending on context [1,2]. Compared with children who use hearing aids or communicate exclusively through sign language, those with CI represent a distinct subgroup whose communication experiences are uniquely influenced by both technological and environmental factors. Although CIs provide significant access to auditory input, integration into mainstream education and social life remains highly variable, influenced by factors such as age at implantation, linguistic exposure, quality of rehabilitation, and the availability of communication support [3,4].

Language development in CI users is shaped not only by auditory experience but also by environmental and sociocultural inputs. Some children achieve near-native spoken language proficiency, while others rely on sign language or a combination of speech and sign, often described as bimodal communication, referring specifically to the concurrent or alternating use of sign and spoken language [5]. Bimodal communication can involve simultaneous use or flexible switching between modalities depending on situational demands. In multilingual societies, these dynamics are further complicated, as school-age children with CIs must navigate not only modality shifts but also interactions across multiple spoken languages [6,7]. Contexts such as multicultural societies in the middle east, including the United Arab Emirates (UAE), exemplify this linguistic diversity, where Arabic and English are widely spoken, and an Arabic sign language variety is used in educational settings that support children with hearing loss. These environments reflect broader patterns observed in other multilingual regions worldwide, where CI users often move fluidly between spoken and signed modalities across different languages. While CI open pathways to spoken language acquisition, many users still encounter barriers such as increased listening effort, difficulty perceiving speech in noise, and challenges with peer inclusion [8,9].

Educational settings present particular challenges. Inadequate classroom acoustics and background noise are widely recognised to affect all learners, yet they have a disproportionately greater impact on school-age children with CI, who depend on optimal listening conditions for speech perception and participation [10,11]. Overlapping speech, reverberation and rapid turn-taking can increase listening effort even when assistive technologies are used [12]. Beyond the classroom, peer and family communication patterns strongly influence the academic and social inclusion of school-age children with CI, which together contribute to the overall success of school-age CI users. Household language practices whether sign-dominant, spoken-dominant, or bimodal strongly influence whether school-age children with CI favour spoken, signed, or bimodal strategies [8,13].

Despite growing literature on CI outcomes, much research remains centred on clinical and linguistic outcomes often drawn from monolingual, western populations [5,6,14,15]. Although studies from multilingual or non-western contexts are emerging, they remain comparatively limited and often address linguistic proficiency rather than everyday communication experiences. As a result, less is known about how school-age children with CI navigate communication in diverse sociolinguistic environments, where the ability to flexibly use spoken and signed modalities is increasingly viewed as essential for academic participation and social inclusion. Understanding these patterns within multilingual settings such as the UAE provides insight into challenges and facilitators that are likely shared across other culturally diverse societies. The UAE represents a linguistically diverse context in which Arabic and English are widely spoken, and Arabic Sign Language is used in educational and social settings supporting children with hearing loss.

Building on the multilingual and bimodal communication context of the UAE, where Arabic, English and Arabic Sign Language are routinely used, this study explores how such environments shape communication preferences among school-age children with CI. It examines whether these school-age children with CI predominantly rely on spoken language, sign language, or bimodal strategies, and explores how these preferences are shaped by home language, adaptability (self-reported flexibility) and perceived effectiveness. Interpreted within a global public-health framework that emphasises equitable access to communication and learning, the findings provide evidence to inform inclusive-education and hearing-health policy by identifying how environmental and linguistic factors influence participation among school-age children with CI.

## 2. Materials and Methods

### 2.1. Study Design

This study employed a descriptive cross-sectional design. Quantitative and qualitative data were collected between 13 January and 25 February 2025 using a structured questionnaire administered to school-age children with CI enrolled in educational programme at Al Amal School for the Deaf, Sharjah City for Humanitarian Services (SCHS), UAE. Al Amal is a bilingual (sign and spoken) educational setting within SCHS that provides academic instruction and speech–language support for children with hearing loss. The study was conducted within a multilingual educational environment where Arabic, English, and Arabic Sign Language are routinely used, although multilingualism was not analysed as a separate study variable, it represents an inherent contextual feature of the participants’ communication environment. Recruitment was facilitated through class teachers, who contacted all eligible students and assisted with the parental consent process. Participation was voluntary, and written parental consent was obtained for all participants and verbal assent was obtained from the school-age children with CI prior to data collection. Ethical approval was granted by the Research and Ethics Committee [Ref/78/2025], in accordance with the principles of the Declaration of Helsinki.

### 2.2. Study Participants

A total of 32 school-age children with CIs participated. All participants used at least one CI. Purposive sampling was used to ensure representation across age, gender, and educational backgrounds. Inclusion criteria were: (1) aged 5 to 18 years, corresponds to the full enrolment spectrum at SCHS schools; (2) use of a CI; and (3) active enrolment in an SCHS educational programme. Exclusion criteria were: (1) absence of a CI and (2) additional disabilities that prevented independent completion of the questionnaire. SCHS is among the largest centres for children with hearing loss in the UAE, providing a concentrated and accessible cohort of CI users. Although modest in size, the sample is consistent with other exploratory studies involving paediatric CI users [5,8] and demonstrates the feasibility of accessing this population within a specialised educational setting.

Participants were enrolled in inclusive educational environments at SCHS, where instruction integrates spoken language and sign-supported modalities. Classrooms included both deaf and hard-of-hearing students, with teachers using mixed communication approaches based on students’ needs. Families of participants were primarily hearing, and home communication ranged from spoken language only to bimodal (spoken and sign) interaction.

### 2.3. Questionnaire Development

The questionnaire was developed following a literature review of communication preferences and language use among children with CI [1,2,5]. Specifically, the reviewed studies provided conceptual frameworks for classroom and bilingual communication themes [1,2] and contextual evidence on how bilingual environments influence communication outcomes [5]. It was drafted in English, translated into Arabic and underwent forward and backward translation to ensure conceptual and linguistic equivalence. Content validity was established through review by a panel of audiologists and speech-language pathologists experienced in CI rehabilitation. Participants were also asked to reflect on their perceived flexibility in switching between spoken and sign communication across settings, which was treated descriptively as self-reported adaptability. The final questionnaire comprised four sections:

- Demographics: age, gender, grade, duration of CI use.
- Communication Preferences: reported use of spoken language, sign language, or bimodal strategies across academic and social contexts.
- Perceived Effectiveness and Comfort: ratings of ease and effectiveness of each mode.
- Qualitative Insights: open-ended questions on communication challenges and suggestions for improvement.

Response formats included multiple-choice,5- point Likert scale (1 = Very Uncomfortable to 5 = Very Comfortable), and open-text items. The complete instrument is available in Supplementary Material S1.

### 2.4. Pilot Testing and Validation of Questionnaire

Face and content validity were ensured through expert review by the same panel of two audiologists and two speech-language pathologists. The instrument was then piloted with eight school-age children with CI (4 males, 4 females; mean age = 12.8 years, SD = 2.3), representing a similar demographic distribution to the main cohort. These participants were excluded from the final sample. Internal consistency across Likert-scale items yielded a Cronbach’s alpha of 0.78, indicating acceptable reliability. Test–retest reliability for categorical items was assessed with a two-week interval in the pilot sample, showing substantial agreement (Cohen’s kappa = 0.73). These values should be interpreted cautiously given the small pilot group but provide preliminary evidence supporting the instrument’s reliability in this population.

### 2.5. Data Analysis

Descriptive statistics summarised demographic characteristics and communication preferences. Associations between categorical variables were analysed using Chi-squared or Fisher’s exact as appropriate. Relationships between continuous variables were examined using Pearson’s correlation. Given the modest sample, all analyses were considered exploratory, and results interpreted with caution.

### 2.6. Qualitative Analysis

Open-ended responses were analysed using Braun and Clarke’s six-step thematic analysis framework (familiarisation, coding, theme generation, review, definition, and reporting) [16]. Although the open-ended questions were aligned with the study objectives, coding was conducted inductively, allowing themes to emerge directly from participants’ responses without pre-imposed categories. Two authors (MA and MM) independently coded the data and discussed discrepancies until consensus was reached, achieving high initial inter-coder agreement. Representative quotations were selected to illustrate key themes.

## 3. Results

### 3.1. Participant Characteristics

Thirty-two school-age children with CI participated in the study. The mean age was 13.4 years (SD = 2.4; range 9–18), with 20 males (62.5%) and 12 females (37.5%). CI configuration included 25 unilateral and 7 bilateral users. The mean duration of CI use was 11.0 years (SD = 2.9; median 10). Participants represented diverse language exposure backgrounds: 53.1% used both spoken and sign language, 28.1% used spoken language only, and 18.8% used sign language only, regularly switching between modalities across home and social settings.

### 3.2. Communication Preferences Across Contexts

**Figure 1:**
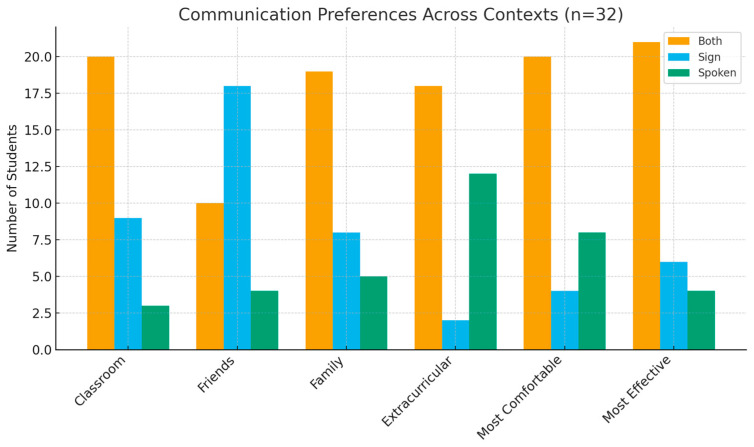
Communication Preferences Across Contexts.

As shown in Figure 1, bimodal communication (use of both spoken and sign language) was the predominant strategy across all contexts. More than half of the children reported using both modes in classroom, family, and extracurricular settings, while exclusive reliance on sign or spoken language was less common. Similar patterns were observed for perceived comfort and effectiveness, with the majority identifying bimodal communication as the most comfortable and effective mode.

### 3.3. Associations with Demographic Factors and Adaptability

Significant associations were found between home language and communication preferences across classroom, family, and extracurricular settings, with medium-to-large effect sizes (Cramer’s V = 0.46–0.75) (Table 1). A gender difference was also observed in family communication (*p* = 0.036, V = 0.46), while no associations emerged for age or grade level.

Adaptability was self-reported by 73% of respondents (19 of 26 valid responses) as “often” or “always” switching between communication modes. Given its relevance to flexible communication across contexts, adaptability was analysed separately to determine potential associations with demographic variables. No significant associations were observed between adaptability and age group, grade level, or classroom communication preference (Table 2).

### 3.4. CI Duration and Comfort Levels

Pearson’s correlation showed a modest positive correlation between CI duration and comfort with spoken communication (r = 0.32, *p* = 0.038). No significant correlation was observed for sign comfort (r = 0.12, *p* = 0.472). The modest effect sizes highlight the exploratory nature of these findings.

### 3.5. Thematic Analysis of Open-Ended Responses

Three key themes emerged from the inductive analysis of open-ended responses. A total of 23 participants provided qualitative comments, generating 38 short statements across the three open-ended questions. Most responses were one or two brief sentences, and several participants addressed more than one topic.

1. Spoken communication challenges (*n* = 14): Participants reported that spoken language was more difficult than sign in noisy or fast-paced situations (e.g.,“صعبة غير لغة الإشارة/Hard not like sign language”).
2. Social barriers to sign use (*n* = 11): Participants emphasised that peers and teachers often lacked sign knowledge (e.g., “عدم معرفة الناس للغة الإشارة/Not all people know sign language”).
3. Recommendations for improvement (*n* = 8): Participants suggested wider public use of sign language and increased interpreter availability (e.g., “لغة الإشارة منتشر قليلا/sign language not public”; “مترجم قليلون/fewer interpreters”).

The analysis reflects a concise but consistent dataset in which recurring experiences, rather than extended narratives, were reported. Responses were categorised according to semantic similarity, and statements that spanned multiple issues contributed to more than one theme. Themes were descriptive, without causal interpretation. These qualitative responses complement the quantitative results, illustrating how participants used bimodal communication within their everyday environments.

## 4. Discussion

This study examined the communication preferences of school-age children with CIs in a multilingual educational and social environment in the UAE, where Arabic, English, and sign language are routinely used. Most participants reported adopting bimodal communication, alternating between spoken and sign language across classroom, family, friends, and extracurricular contexts. This strategy was described as both comfortable and effective, supporting the view that bimodal use is a functional default rather than a compensatory response [17,18]. These findings emphasise that communication outcomes are shaped not only by auditory access but also by the social and educational environments in which children participate, linking clinical audiology with broader issues of inclusion and public health.

Our results align with an expanding literature on bimodal bilingualism in children with CIs. Goodwin et al. demonstrated that bimodal bilingual CI users achieved spoken language accuracy comparable to hearing peers without compromising sign proficiency [17]. Similarly, Geers et al. reported that early-implanted children exposed to sign still developed strong spoken skills, challenging assumptions that sign hinders oral outcomes [19]. Together, these findings suggest that exposure to and support for both spoken and sign modalities can enhance children’s communicative flexibility and participation within educational and social settings. Creating environments that value and facilitate both modalities may therefore strengthen inclusion and learning outcomes for school-age CI users.

A critical determinant in our study was the home language environment. School-age children with CI from families who used both spoken and sign languages were significantly more likely to extend this practice into academic and social contexts, while those from sign dominant households remained more sign focused. This supports evidence that family engagement and early bilingual exposure strongly predict developmental outcomes [8]. Systematic reviews confirm that children whose families provide rich input in both modalities achieve superior outcomes across language domains [20], and further studies highlight the role of supportive family environments in fostering linguistic flexibility and cognitive growth [21].

Collectively, these findings underscore the importance of family-centred guidance and support strategies that help caregivers incorporate bimodal communication into daily routines. In multilingual and multicultural contexts such as the UAE, such approaches may require adaptation to reflect families’ language resources and cultural practices. At a systems level, early and rich exposure to both spoken and signed modalities should be recognised as a determinant of equitable developmental outcomes, consistent with priorities in education and global public health policy [22]. Further research is needed to examine how these family-centred strategies can be effectively implemented in multilingual environments.

Gender differences, though limited to home settings, were observed, with male participants reporting greater reliance on sign than females. The literature remains inconsistent: some evidence suggests that parental beliefs and sociocultural expectations can influence modality use in ways that indirectly reflect gendered patterns [23]. Large-scale outcome studies, however, have not reported systematic gender differences in early CI outcomes [24,25]. Given the modest sample size, this result should be seen as exploratory. It highlights how gender norms can intersect with disability and communication practices. This intersection has important implications for both education and public health, where equity and inclusion depend on recognising such social influences.

Another notable result was the discrepancy between perceived adaptability and measured associations. Nearly three quarters of school-age children reported switching between communication modes depending on context, yet statistical analyses did not confirm significant associations between adaptability and classroom preferences. Similar gaps between self-reported adaptability and observed practice have been documented elsewhere [26,27]. This suggests that adaptability may reflect how school-age children wish to see themselves rather than a skill they consistently apply or that it shows up more often in informal settings than in structured classrooms. Within multilingual contexts such as the UAE, adaptability may also involve navigating multiple spoken languages alongside sign, adding cognitive and linguistic demands. Rehabilitation should therefore go beyond modality training to support applied adaptability, ensuring that self-reported flexibility translates into observable behaviour.

The preference for bimodal communication in this cohort likely reflects both need and opportunity [17,18,28]. School-age children rely on both modalities not only to overcome auditory challenges but also because environmental supports, such as interpreter availability and sign-fluent peers, make bimodal use more feasible. This highlights that communication access is influenced by broader structural determinants, including school resources, family practices, and community inclusion rather than being solely an individual choice. Recognising communication access as a social and environmental determinant of participation aligns with global frameworks on inclusive education and health equity [22,29]. Thus, recognising bimodal communication within this perspective strengthens its relevance for inclusive education and public health policy, where communication access is recognised as a determinant of equity.

School-age children in this study had a mean of 11 years of CI use, which may help explain their relatively high levels of comfort in spoken communication. However, causality cannot be inferred in a cross-sectional design. Longitudinal evidence consistently shows that earlier activation and longer CI duration are associated with stronger spoken outcomes [18]. More recent studies also demonstrate that children implanted early and supported by engaged families achieve better long-term competence [15,30]. Our data provide descriptive support for this pattern, underscoring the need for sustained follow-up into school-age and adolescence. This echoes public health principles of continuity of care and life-course approaches to rehabilitation, which emphasise ongoing support beyond early childhood to sustain participation and well-being across developmental stages [22,31].

Taken together, these findings reinforce the consensus that CI outcomes cannot be reduced to speech perception scores alone. Communication is increasingly recognised as a multimodal, socially situated process shaped by environmental, familial, and cultural determinants alongside auditory access [30,32,33]. By drawing attention to a multilingual context, this study adds to a literature base dominated by Western, monolingual samples [19,25], and emphasises that inclusive policies must address linguistic diversity as well as auditory access. From a public-health perspective, communication access contributes to inclusion and equitable participation. Integrating hearing-health awareness within inclusive education systems and expanding service delivery such as interpreter provision, sign language training and family-centred rehabilitation may strengthen equity and long-term well-being among school-age children with CI [34].

The qualitative findings add nuance by highlighting lived experiences. School-age children reported spoken language as especially challenging in noisy or fast-paced environments, consistent with evidence on listening effort [23,33]. They also identified social barriers, particularly peers and teachers limited sign knowledge, which led to exclusion. Importantly, participants proposed solutions such as wider public use of sign and greater interpreter availability. These perspectives illustrate that communication barriers are not only technical but also social and structural, underscoring the need for policy-level action in line with disability inclusion frameworks [34].

### 4.1. Public Health and Educational Implications

The findings have important implications for inclusive education and public health. Schools may benefit from incorporating bimodal bilingual approaches, including interpreter provision, sign language training for staff, and peer awareness programs. Such measures can reduce listening effort, improve access to academic content, and promote peer inclusion. While the present study did not directly measure listening effort; existing evidence suggests that reducing communication barriers and multimodal instructional strategies can mitigate cognitive effort and enhance engagement in classroom interactions [12,35]. Clinicians should prioritise family-centred interventions, equipping caregivers with bilingual strategies such as sign instruction, home-based communication coaching, and dual-language literacy support. Rehabilitation programmes should also include adaptability training, enabling children to practice switching modalities in authentic, real-world contexts.

From a public health perspective, communication access should be recognised as a contributor to inclusion and equitable participation. Achieving equitable participation for CI school-age children requires policies that integrate hearing health into inclusive education systems, promote sign language awareness in the wider community, and sustain rehabilitative support across developmental stages. These findings align with broader calls for disability inclusive education as a public health priority, linking communication access to long-term outcomes in learning, social participation, and mental health [22,31,34].

### 4.2. Limitations

Several limitations must be acknowledged. The sample size limits generalisability and statistical power, restricting the scope for multivariate modelling. Reliance on self-reported measures may have introduced bias, especially in reported adaptability. The cross-sectional design precludes causal inferences about the role of CI duration. Detailed clinical variables, such as device make or model, speech-processor settings, mapping parameters, and auditory thresholds, were not systematically collected, which limits interpretation of individual variability in communication outcomes. Contextual variables such as socio-economic status, quality of teaching, and rehabilitative resources were not included, limiting the analysis of structural determinants. As this exploratory design identified what communication patterns occur among school-age children with CI rather than how these patterns develop, future research should examine the underlying processes across multilingual and monolingual contexts. Future tool development should include CI users or caregivers to further enhance ecological validity

### 4.3. Future Directions

Future research should employ longitudinal and multi-site designs across different educational settings within and beyond the UAE to capture developmental trajectories and cross-cultural variations. Larger samples would allow for multivariate models that test interactions between demographic, linguistic, and social determinants. Intervention studies could evaluate the impact of parent coaching, adaptability training, and bimodal curricula on communication outcomes. Further, linking communication preferences with cognitive development, psychosocial well-being, and academic achievement would provide deeper insights. Policy-oriented research should explore how interpreter provision, teacher training, and disability inclusion frameworks can be integrated into school and community systems.

## 5. Conclusions

This study suggests that school-age children with CI in multilingual environments predominantly adopt bimodal communication, which appears related to home-language patterns and sociocultural influences. These findings may have implications for inclusive frameworks spanning education, rehabilitation, and public health. Recognising bimodal communication as a contextually normative practice, rather than compensatory one, may enhance language access, promote equity in learning, and strengthen social integration for school-age children with CI.

## Figures and Tables

**Table 1:** Associations Between Communication Preferences and Demographics.

Tested Association	χ^2^ (df)	*p*-Value	Effect Size
Gender (family)	6.67 (2)	0.036	Cramer’s V = 0.46
Home language (classroom)	13.67 (4)	0.008	V = 0.46
Home language (family)	36.00 (4)	<0.001	V = 0.75
Home language (extracurricular)	22.38 (4)	<0.001	V = 0.59
Home language (Friends)	10.65 (4)	0.031	V = 0.41
Age group (comparative analysis only)	5.12 (4)	0.276	V = 0.25
Grade level (comparative analysis only)	4.67 (4)	0.322	V = 0.23

Note: Percentages are of non-missing responses; rows may not sum to 100 due to rounding. Tests conducted using Chi-square or Fisher’s exact (when expected cell counts < 5). Effect sizes reported as Cramer’s V.

**Table 2:** Associations Between Self-Reported Adaptability and Demographic Variables.

Tested Association	χ^2^ (df)	*p*-Value	Effect Size
Adaptability vs. classroom preference	1.54 (2)	0.463	V = 0.24
Adaptability vs. age group	2.03 (2)	0.362	V = 0.28
Adaptability vs. grade level	1.45 (2)	0.484	V = 0.22

Note: Tests conducted using Chi-square or Fisher’s exact (when expected cell counts < 5). Effect sizes reported as Cramer’s V.

## Data Availability

The datasets used and analysed in the current study can be obtained from the corresponding author, subject to reasonable request.

## Supplementary Materials

The following supporting information can be downloaded at: https://www.mdpi.com/article/10.3390/ijerph22111699/s1. Questionnaire: Diverse Communication Preferences of school-age children with Cochlear Implants.

## Abbreviations

The following abbreviations are used in this manuscript:

CI	Cochlear Implants
UAE	United Arab Emirates
SCHS	Sharjah City for Humanitarian Services
