# Supplementary material for: Communication Preferences of School-Age Children with Cochlear Implants in Multilingual Educational Settings: Implications for Inclusive Education and Public Health
Source: Int J Environ Res Public Health. 2025 Nov 11;22(11):1699. doi: 10.3390/ijerph22111699 (PMC12652520; doi:10.3390/ijerph22111699)
Supplement: Supplementary file 1 [file ijerph-22-01699-s001.zip › ijerph-3916833-supplementary.pdf]

Diverse Communication Preferences of School Age with Cochlear Implants:

Study Questionnaire:

Section 1: Demographic Information

1. Age:
2. Gender: Male / Female
3. Grade/Class:
4. Duration of CI Use: _____ years
5. Age at which hearing loss identified:
6. History of hearing loss in the family: Parents/ siblings:
7. Primary Language at Home: Spoken Language / Sign Language / Both

Section 2: Communication Preferences in Different Settings

8. Which mode of communication do you prefer during lessons in the classroom?
   a) Spoken Language
   b) Sign Language
   c) Combination of Both
9. Which mode of communication do you use most often with friends during breaks?
   a) Spoken Language
   b) Sign Language
   c) Combination of Both
10. Which mode of communication do you use with your family?
    a) Spoken Language
    b) Sign Language
    c) Combination of Both
11. Which mode of communication do you prefer during extracurricular activities?
    a) Spoken Language
    b) Sign Language
    c) Combination of Both
12. When learning new concepts, which mode of communication helps you understand better?
    a) Spoken Language

b) Sign Language

c) Combination of Both

13. When asking questions to the teachers, which mode of communication do you prefer to use?

a) Spoken Language

b) Sign Language

c) Combination of Both

Section 3: Perceived Effectiveness and Comfort

14. Which mode of communication do you feel most comfortable using?

a) Spoken Language

b) Sign Language

c) Combination of Both

15. Which mode of communication do you think is most effective for you in understanding and being understood?

a) Spoken Language

b) Sign Language

c) Combination of Both

16. Do you switch between communication modes depending on the situation?

a) Yes

b) No

c) If yes, when and why do you switch? (Open-ended)

17. Rate your comfort level using spoken language in different settings

(1 = Very Uncomfortable, 5 = Very Comfortable):

**Settings**	**1 (Very uncomfortable)**	**2 (Uncomfortable)**	**3 (Neutral)**	**4 (Comfortable)**	**5 (Very Comfortable)**
**In the classroom**					
**With friends during breaks**					
**With family**					
**During extracurricular activities**					

18. Rate your comfort level using sign language in different settings

(1 = Very Uncomfortable, 5 = Very Comfortable):

Settings	1 (Very uncomfortable)	2 (Uncomfortable)	3 (Neutral)	4 (Comfortable)	5 (Very Comfortable)
**In the classroom**					
**With friends during breaks**					
**With family**					
**During extracurricular activities**					

Section 4: Open-Ended Questions

19. What challenges do you face when using spoken language?

20. What challenges do you face when using sign language?

21. Do you have any suggestions to make communication easier for you?

تفضيلات التواصل المتنوعة للطلاب المستخدمين لزراعة القوقعة الصناعية:

الباحثون: محمد اياس[1]، مروه عبد الفتاح ماضي[2]
الجهة: 1-جامعة الشارقة، الامارات العربية المتحدة، 2- مدينة الشارقة للخدمات الإنسانية

هدف الدراسة: تحديد تفضيلات الأطفال لأنماط التواصل المختلفة (اللغة المنطوقة، لغة الإشارة أو مزيج من الاثنين معا) في البيئات المختلفة كمستخدمين لزراعة القوقعة الصناعية.

المشاركون في الدراسة: الاطفال مستخدمي زراعة القوقعة الصناعية في مدرسة الصم.

طريقة الدراسة: سيتم تقديم استبيان مخصص باللغتين العربية والإنجليزية لجمع البيانات حول تفضيلات التواصل لدى الاطفال مستخدمي زراعة القوقعة الصناعية

استبيان الدراسة

القسم 1: المعلومات الديموغرافية

1. العمر:
2. الجنس: ذكر/أنثى
3. الصف :
4. مدة استخدام جهاز زراعة القوقعة: ــــــ سنة
5. العمر الذي تم فيه اكتشاف فقدان السمع:
6. وجود تاريخ لفقدان السمع في العائلة (الأهل/ الأشقاء) :
7. اللغة الأساسية في المنزل
   أ) اللغة المنطوقة
   ب) لغة الإشارة
   ج) كلاهما

القسم 2: تفضيلات التواصل في البيئات المختلفة

8. ما هو أسلوب التواصل الذي تفضله أثناء الحصة الدراسية في الغرفة الدراسية؟

أ) اللغة المنطوقة

ب) لغة الإشارة

ج) الجمع بين الاثنين

9. ما هو أسلوب التواصل الذي تستخدمه غالبًا مع الأصدقاء أثناء فترات الراحة؟

أ) اللغة المنطوقة

ب) لغة الإشارة

ج) الجمع بين الاثنين

10. ما هو أسلوب التواصل الذي تستخدمه مع عائلتك؟

أ) اللغة المنطوقة

ب) لغة الإشارة

ج) الجمع بين الاثنين

11. ما هو أسلوب التواصل الذي تفضله أثناء الأنشطة اللامنهجية؟

أ) اللغة المنطوقة

ب) لغة الإشارة

ج) الجمع بين الاثنين

12. عند تعلم مفاهيم جديدة، ما هو أسلوب التواصل الذي يساعدك على الفهم بشكل أفضل؟

أ) اللغة المنطوقة

ب) لغة الإشارة

ج) الجمع بين الاثنين

13. عند طرح الأسئلة على المعلمين، ما هو أسلوب التواصل الذي تفضل استخدامه؟

أ) اللغة المنطوقة

ب) لغة الإشارة

ج) الجمع بين الاثنين

القسم 3: الفعالية والراحة

14. ما هو أسلوب التواصل الذي تشعر براحة أكبر عند استخدامه؟

أ) اللغة المنطوقة

ب) لغة الإشارة

ج) الجمع بين الاثنين

15. ما هو أسلوب التواصل الذي تعتقد أنه الأكثر فعالية بالنسبة لك في الفَهم والفِهم؟

أ) اللغة المنطوقة

ب) لغة الإشارة

ج) الجمع بين الاثنين

16. هل تقوم بالتبديل بين أساليب التواصل حسب الموقف؟

أ) نعم

ب) لا

ج) اذا كانت الاجابة بنعم ، متى ولماذا يتم التبديل ؟(اجابة مفتوحة)

17. قم بتقييم مستوى راحتك باستخدام اللغة المنطوقة في بيئات مختلفة (1 = غير مريح للغاية، 5 = مريح جدًا):

البيئة	1 (غير مريح للغاية)	2 (غير مريح)	3 (محايد)	4 (مريح)	5 (مريح جدا)
الغرفة الصفية					
مع الأصدقاء اثناء الاستراحة					
مع العائلة					
خلال الأنشطة اللامنهجية					

18. قم بتقييم مستوى راحتك باستخدام لغة الإشارة في بيئات مختلفة (1 = غير مريح للغاية، 5 = مريح جدًا):

البيئة	1 (غير مريح للغاية)	2 (غير مريح)	3 (محايد)	4 (مريح)	5 (مريح جدا)
الغرفة الصفية					
مع الأصدقاء اثناء الاستراحة					
مع العائلة					
خلال الأنشطة اللامنهجية					

القسم الرابع: الأسئلة المفتوحة

19. ما هي التحديات التي تواجهها عند استخدام اللغة المنطوقة؟

20. ما هي التحديات التي تواجهك عند استخدام لغة الإشارة؟

21. هل لديك أي اقتراحات لتسهيل التواصل معك؟
